# Functional imaging analyses reveal prototype and exemplar representations in a perceptual single-category task

**DOI:** 10.1038/s42003-022-03858-z

**Published:** 2022-09-01

**Authors:** Helen Blank, Janine Bayer

**Affiliations:** Department of Systems Neuroscience, University Medical Centre Hamburg-Eppendorf, Martinistr. 52, 20246 Hamburg, Germany

**Keywords:** Perception, Human behaviour, Neural encoding

## Abstract

Similarity-based categorization can be performed by memorizing category members as exemplars or by abstracting the central tendency of the category – the prototype. In similarity-based categorization of stimuli with clearly identifiable dimensions from two categories, prototype representations were previously located in the hippocampus and the ventromedial prefrontal cortex (vmPFC) and exemplar representations in areas supporting visual memory. However, the neural implementation of exemplar and prototype representations in perceptual similarity-based categorization of single categories is unclear. To investigate these representations, we applied model-based univariate and multivariate analyses of functional imaging data from a dot-pattern paradigm-based task. Univariate prototype and exemplar representations occurred bilaterally in visual areas. Multivariate analyses additionally identified prototype representations in parietal areas and exemplar representations in the hippocampus. Bayesian analyses supported the non-presence of prototype representations in the hippocampus and the vmPFC. We additionally demonstrate that some individuals form both representation types simultaneously, probably granting flexibility in categorization strategies.

## Abstract (web summary)

Model-based univariate and multivariate analyses of fMRI data from 62 healthy participants in a dot-pattern paradigm-based task provide further insight into the neural basis of similarity-based categorization.

## Introduction

Categorisation is a fundamental cognitive process that enables us to structure the world, apply prior knowledge to new situations, and thereby facilitate perception. In everyday life, we categorise various stimulus types into different category structures, often without being aware of the categorising process itself, for example, when we recognise faces, emotional expressions, or speech^1–3^. According to prototype and exemplar theories, categorisation can be either performed by representing category knowledge in the form of its central tendency, the ‘prototype’, or by storing category ‘exemplars’ as separate instances^4,5^. Correspondingly, the categorisation of a new item can either be based on its similarity to the prototype or the summed similarities to the exemplars.

What is known about the localisation of prototype and exemplar representation in the brain stems from a few studies using stimuli with clearly identifiable, discrete dimensions (e.g., foot or body shape in imagery birds) and two contrasting categories (A/B tasks), i.e., a particular type of similarity-based categorisation^6–8^. In these studies, exemplar representations were identified in the posterior occipital cortex, the inferior frontal gyrus, and the lateral parietal cortex^6,8^, areas known to typically mediate basic visual memory of single items^9^. Prototype representations were located in the anterior hippocampus and the ventromedial prefrontal cortex (vmPFC)^6,7^. Well-grounded on current knowledge about hippocampal functions beyond episodic memory^6,10,11^, it has been proposed that the interplay between hippocampus and vmPFC mediates prototype abstraction. In everyday life, we frequently discriminate a single category from other stimuli, e.g., when recognising a face as familiar, a fruit as edible, or an animal as dangerous. Therefore, it is an important question whether the findings from two contrasting categories (A/B tasks) can be generalised to single-category tasks (i.e. ‘A/non-A’ problems) in which category membership is defined by the holistic, overall perceptual similarity among category members^12^. In the laboratory, this categorisation problem is often operationalized by the dot-pattern paradigm^13^. While declarative memory systems are supposed to support A/B categorisation of stimuli composed of clearly distinguishable feature dimensions, A/notA categorisation of stimuli like dot patterns is rather supported by the perceptual learning system or striatal learning^14–18^. While this is in line with relatively preserved categorisation performance for perceptual single-category tasks in patients with memory dysfunction and hippocampal lesions^19–22^, the questions arise if and where prototypes are represented in these tasks.

On the behavioural level, an important contribution to the debate over which of the two accounts (i.e., the exemplar or the prototype theory) explains similarity-based categorisation was the finding that individuals can engage both strategies^7,16,23–25^. The additional observation of both prototype, as well as exemplar representations in group-level functional magnetic resonance imaging (fMRI) analyses triggered the idea that individuals could form both representation types simultaneously^11^, entailing the potential to flexibly adapt strategy choices to situational requirements. Nevertheless, the fact that the co-occurrence of both representation types has not yet been investigated in single individuals motivates the question of whether the co-occurrence of both strategy representations was caused by the presence of subgroups with either prototype or exemplar representations or by concurrent representations of both strategies within individuals. Furthermore, it is unclear whether the presence of neural prototypes or exemplar representations is related to behavioural preferences of the corresponding strategy.

**Fig. 1:**
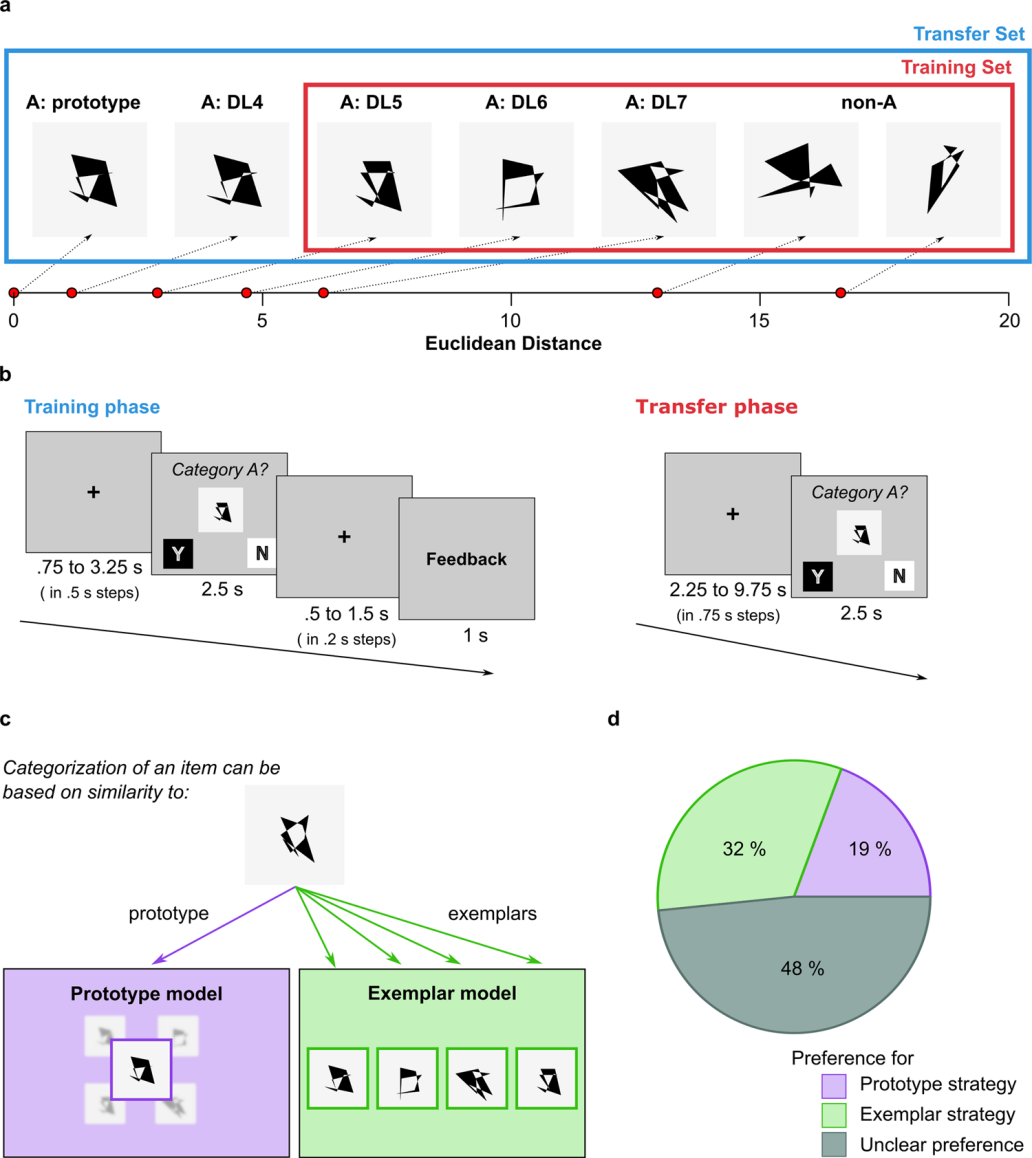
Category learning task and behavioural results. **a** Examples for members of category A for each distortion level (DL) and non-members. The red frame illustrates that only medium to highly distorted category members and non-members were part of the training set. The blue frame illustrates that the transfer set consisted of a full range of stimuli. **b** Trial timing of the training and the transfer phase. Single training blocks had a duration of 2.8 min. Single transfer blocks had a duration of 4.8 min. Positions of ‘yes’ (Y) and ‘no’ (N) responses changed pseudo-randomly between left and right to preclude direct category to motor mapping^94^. **c** Conceptual illustration of the prototype and the exemplar model. The prototype model (purple) assumes that a category is represented as its central tendency, the prototype. The exemplar model (green) posits that a category is represented by storing individual category members as separate instances. Once category representations are established, the category membership of a new item can be determined by comparing its appearance to the representation of the prototype or to the stored exemplars. **d** Behavioural strategy preference. Percentages of volunteers (*n* = 62) showing behavioural preferences for the prototype strategy (purple), the exemplar strategy (light green) and those who showed unclear preferences (dark turquoise).

The primary goal of the current study was to test how the prototype and exemplars are represented in a single-category perceptual categorisation task based on a classical dot-pattern paradigm^13^ (Fig. 1a–c) by combining formal modelling of individual categorisation behaviour with model-based univariate analyses and multivariate representational similarity analyses (RSA) of fMRI data. In addition, we used RSA to test co-existent prototype and exemplar representations on the individual level and their correspondence to individual behavioural strategies.

We demonstrate that prototype representations in a single-category task with abstract visual patterns primarily occurred in visual and parietal but not hippocampal areas or the vmPFC. Exemplar representations were mainly found in visual and memory-related areas like the hippocampus. Moreover, our results indicate that individuals can form both representation types simultaneously.

## Results

### Behavioural results

In the transfer phase, the average accuracy was 0.86 (SD = 0.28) in deciding whether an item belonged to category A or not. Accuracy for category (*M* = 0.85, SD = 0.29) and non-category items (*M* = 0.86, SD = 0.27) did not differ significantly [*F*(1, 79.4) = 0.09, *p* = 0.75] and did not change across blocks [*F*(7, 20118.8) = 0.66, *p* = 0.71]. Among category members, accuracy was significantly predicted by both logarithmized model-free distances to the prototype [*F*(1,19) = 8.28, *p* = 0.010] and to exemplars [*F*(1,19) = 29.14, *p* < 0.001].

Averaged posterior means of the sensitivity parameter *c*, quantifying individual sensitivies to distances from the prototype or exemplars, and the decision criterion parameter *k* were very similar for both models (*M*_*C*_ = 2.55, SD_*C*_ = 0.84 and *M*_*k*_ = 0.038, SD_*k*_ = 0.101 for the prototype and *M*_*C*_ = 2.64, SD_*C*_ = 0.83 and *M*_*k*_ = 0.022, SD_*k*_ = 0.039 for the exemplar model). Neither averaged DIC’s (prototype model: *M*_DIC_ = 7.45; exemplar model: *M*_DIC_ = 4.50) nor a paired *t*-test on individual DIC’s showed a clear preference for one strategy on the group level [*t*(61) = −1.54, *p* = 0.128]. Both models showed clearly better fits than the guessing model (*M*_DIC_ = 53.67; prototype model: *t*(61) = −17.75, *p* < 0.001; exemplar model: *t*(61) = −15.54, *p* < 0.001). There was a strong relationship between observed and predicted endorsement rates for both models (*M*_*r*_ = 0.806, SD_*r*_ = 0.121 for the prototype and *M*_*r*_ = 0.815, SD_*r*_ = 0.105 for the exemplar model).

The deviance information criterion (DIC)^26^ was used as a measure of model fit to identify individual strategies. A comparison of individual DICs from the two models suggests that 12 individuals preferred the prototype strategy [i.e., (DIC_exemplar_ – DIC_prototype_) ≥5] and 20 individuals preferred the exemplar strategy [i.e., (DIC_prototype_ – DIC_exemplar_) ≥5], while the remaining 30 volunteers did not show a clear preference [i.e., |(DIC_exemplar_ – DIC_prototype_)| <5; Fig. 1d). There was no indication for significant differences in total accuracy between the three preference groups [*F*(2,59) = 0.37, *p* = 0.692]. All included participants showed better fits of the prototype or the exemplar model than the guessing model.

Please refer to Supplementary Note 1 for analyses of behavioural performance in the training phase.

### Univariate prototype and exemplar representations on the group level

Univariate and searchlight representational similarity analyses (RSA; see below) of fMRI data were used to localise prototype and exemplar representations in the whole brain. In univariate analyses, the application of positive and negative contrasts to the parametric modulators allows for investigating the perceptual similarity (positive contrast) and perceptual dissimilarity (negative contrast) to the prototype and exemplars.

**Fig. 2:**
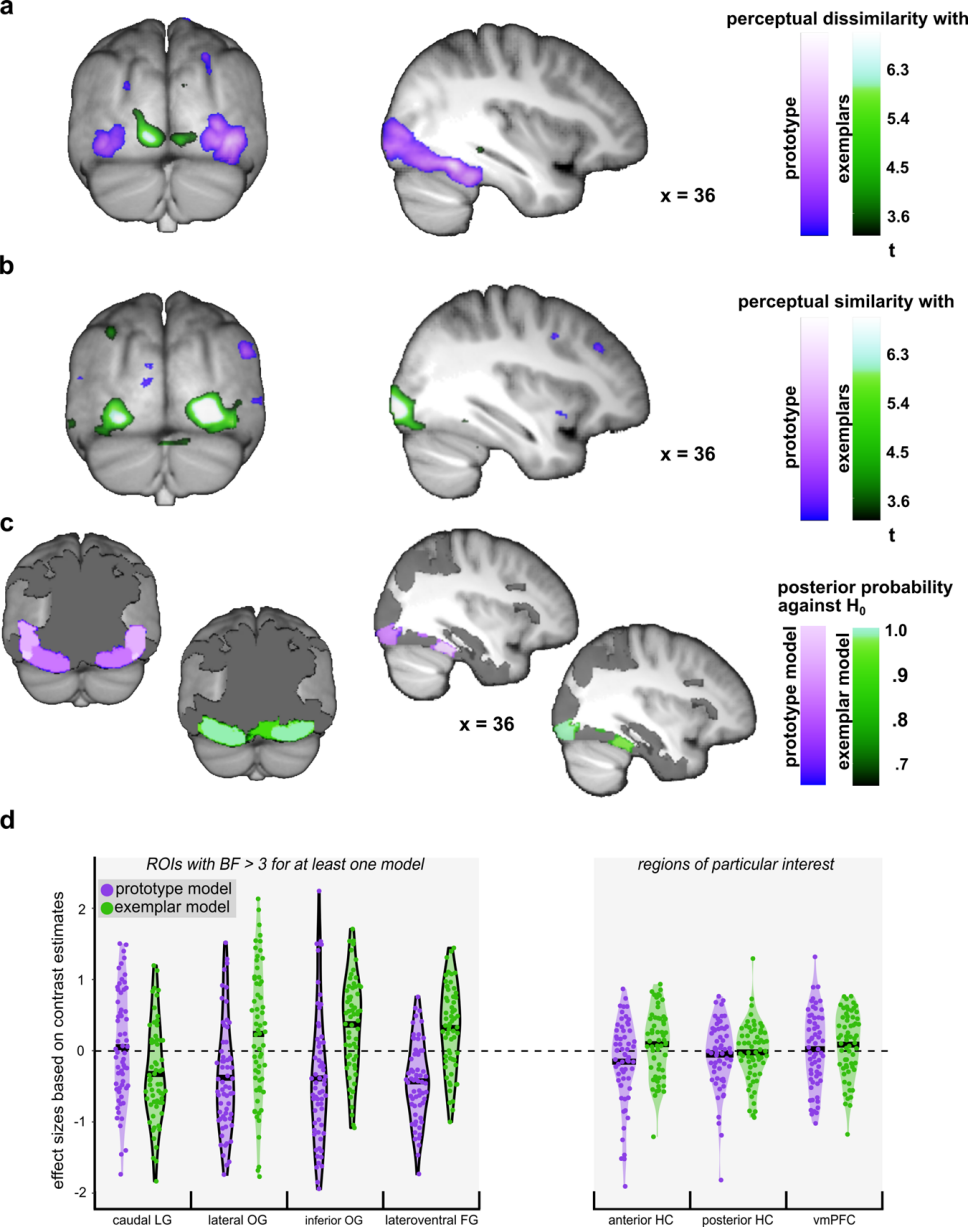
Univariate prototype and exemplar representations (*n* = 62 individuals). **a** Negative correlation with perceptual similarity (i.e. perceptual distance). Regions sensitive to model-based perceptual distances to the prototype (purple) and to exemplars (green). A search depth of 16 voxels was applied to also depict exemplar model representations located slightly below the cortical surface. **b** Positive correlation with a perceptual similarity. Regions sensitive to model-based similarities to the prototype (purple) and to exemplars (green). **a**, **b** Statistical *t*-maps are thresholded at *p*_unc_ **<** 0.001 for visualisation. **c** ROI-specific posterior probabilities that univariate effect sizes (ES_univ_) for prototype (purple) and exemplar (green) representations deviate from zero. Only ROIs with a BF **>**3 for the comparison against the null model are depicted. **d** Violin plots of observed ES_univ_ for ROIs with a BF >3 for the comparison against the null model and regions of particular interest. The bolded horizontal lines depict the mean ES within each ROI. Black borders around the violin plots highlight effects with a BF **>**3.

Perceptual dissimilarity, i.e., negative correlations with perceptual similarity of each category member to the prototype or the exemplars, correlated mainly with activity in visual areas with no evidence for the involvement of higher cognitive areas. In particular, perceptual dissimilarity to the prototype was associated with robust bilateral activations in lower and higher visual processing areas, extending from the inferior and lateral occipital gyri to the right fusiform gyrus (Fig. 2a and Table 1). Perceptual dissimilarity to exemplars was associated with effects in lower visual areas of the left hemisphere, such as the posterior part of the inferior occipital gyrus, extending into the lingual gyri and the cuneus (Fig. 2a and Table 1).

**Table 1:** Localisation of univariate prototype and exemplar representations.

		Peak voxels and local maxima					Cluster extent
		MNI coordinates					
		*x*	*y*	*z*	*Z*	*p*_FWE_	*k*_FWE_
Perceptual distance to prototype							
Medioventral and lateroventral FG	R	38	−52	−18	5.67	0.001	26
Inferior and lateral OG area V5	R	44	−72	−8	5.32	0.006	46
Perceptual distance to exemplars							
Inferior OG, posterior occipital cortex, caudal lingual gyrus, caudal cuneus	L	−12	−92	−6	5.9	<0.001	84
Perceptual similarity to exemplars							
Inferior and middle OG, posterior occipital cortex	R	30	−96	−2	7.33	<0.001	367
		36	−90	−8	6.68	<0.001	
		48	−80	−12	5.25	0.008	
Inferior OG, posterior occipital cortex	L	−30	−96	−10	6.1	<0.001	95

*FG* fusiform gyrus, *OG* occipital gyrus.

Peak voxels and local maxima for the univariate effects of perceptual distance and similarity to the prototype and exemplars surviving family-wise correction for multiple comparisons across the entire scan volume (*p*FWE <0.05, k ≥ 10).

Analyses did not yield significant positive correlations with perceptual similarity to the prototype in any region. Perceptual similarity to exemplars correlated again with activity in lower visual areas such as the bilateral posterior occipital cortex, extending into inferior occipital gyri (Fig. 2b and Table 1).

Bayesian analyses were used to quantify the probability by which univariate effect sizes (ES_univ_) in bilateral anatomical masks deviate from zero (Fig. 2c and Supplementary Table 1). Confirming results from our frequentist analyses, Bayesian analyses provided evidence for the association between univariate activity and (dis-)similarity to the prototype or exemplar representations, respectively. Specifically, Bayesian analyses revealed (1) strong evidence for dissimilarity to the prototype in the lateral occipital gyrus, with moderate evidence for the non-presence of exemplar associations in this region; (2) strong evidence for dissimilarity to exemplars in the caudal lingual gyrus, with very strong evidence for the non-presence of prototype associations, (3) moderate evidence for dissimilarity to the prototype in the inferior occipital gyrus, with very strong evidence for similarity to exemplars; and (4) very strong evidence for dissimilarity to the prototype and for similarity to exemplars in the lateroventral fusiform gyrus (see left column of Fig. 2d for individual effect sizes (ES_univ_) from regions of interest (ROIs) with at least moderate evidence for prototype or exemplar representations). While the first three results from the Bayesian analyses confirm the results from the frequentist analysis, the fourth result adds evidence for univariate exemplar representation in the fusiform gyrus.

In contrast to previously reported univariate prototype representations in the hippocampus for the similarity-based categorisation of stimuli with clearly identifiable dimensions from two categories (A/B tasks)^6,7^, we did not detect significant effects either associated with perceptual dissimilarity (largest effect at *x* = 26, *y* = −28, *z* = −8; *Z* = 3.08, *p*_unc_ = 0.001, *p*_FWEROI_ n.s.) or perceptual similarity to the prototype (largest effect at *x* = −28, *y* = −40, *z* = −2; *Z* = 2.90 *p*_unc_ = 0.002, *p*_FWEROI_ n.s.) in this region. Current data also do not provide evidence for univariate prototype representations in the vmPFC, neither for perceptual dissimilarity (largest effect at *x* = −20, *y* = 20, *z* = −24; *Z* = 2.55, *p*_unc_ = 0.005, *p*_FWEROI_ *n.s*.) nor perceptual similarity (largest effect at *x* = −8, *y* = 22, *z* = −4; *Z* = 2.68, *p*_unc_ = 0.004, *p*_FWEROI_ *n.s*.). The non-presence of univariate exemplar representations was confirmed by strong evidence in the anterior hippocampus and the vmPFC and very strong evidence in the posterior hippocampus (see Fig. 2d for individual effect sizes extracted from hippocampal and vmPFC masks).

### Multivariate prototype and exemplar representations on the group level

**Fig. 3:**
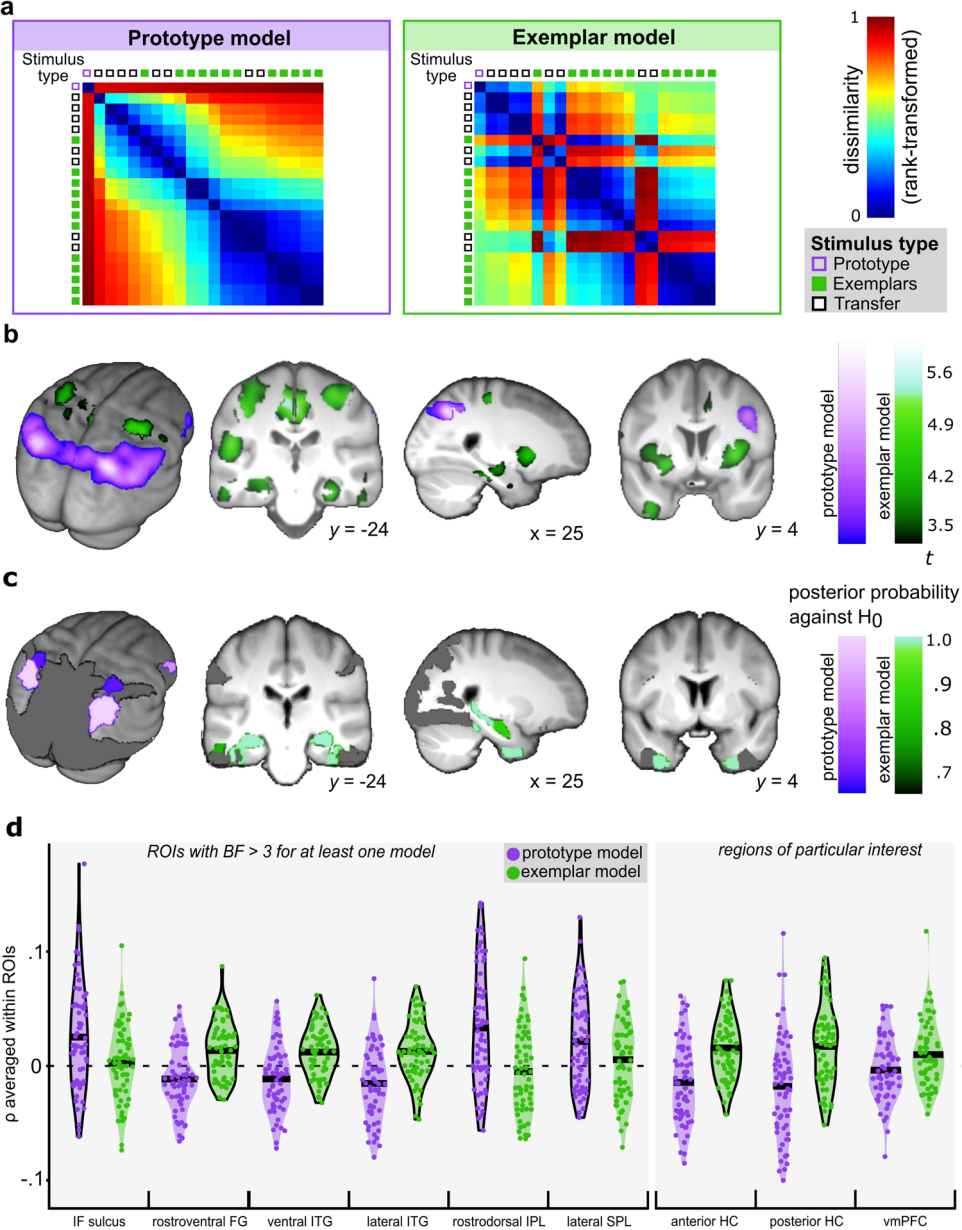
Multivariate analyses on the group level (*n* = 62 individuals). **a** Examples of representational dissimilarity matrices (RDM) for the prototype and the exemplar model from one volunteer. Each cell in the model RDM represents absolute differences between similarity estimates of a category member stimulus pair. For visualisation purposes, both model RDMs were sorted by similarity to the prototype in descending order and were rank-transformed. The small squares represent single stimuli. The prototype, which is also a category A transfer item, is denoted by a purple framed square. All other transfer items are denoted by black framed squares. The exemplars are denoted by green-filled squares. **b** Multivariate prototype and exemplar representations. Multivariate representational similarity analyses (RSA) show significant fits of the prototype (purple) and the exemplar (green) models. Statistical *t*-maps are thresholded at *p*_unc_ **<** 0.001 for visualisation purposes. **c** ROI-specific posterior probabilities that multivariate effect sizes (ES_RSA_) for prototype (purple) and exemplar (green) representations deviate from zero. Only ROIs with a BF **>**3 for the comparison against the null model are depicted. **d** Violin plots of observed ES_RSA_ for ROIs with a BF >3 for the comparison against the null and ROIs of particular interest. The bolded horizontal lines depict the mean ES within each ROI. Black borders around the violin plots highlight effects with a BF **>**3. Black borders around the violin plots highlight effects with a BF **>**3.

To investigate whether a multivariate approach could uncover additional localisations of prototype and exemplar representations in the brain, searchlight RSA comparing fMRI patterns with model representational dissimilarity matrices (RDMs; see Methods Section ‘Multivariate fMRI representational similarity analyses’) based on stimulus similarity to the prototype and exemplars was performed (Fig. 3a).

Multivariate analyses revealed prototype representations in parietal, parieto-occipital and occipito-temporal regions (see Fig. 3b and Table 2), specifically in the intraparietal sulcus [IPS; i.e., bilateral inferior parietal lobule (IPL) and superior parietal lobule (SPL)] and bilateral lateral superior occipital gyri, as well as in the right fusiform and the right inferior temporal gyrus (ITG). In addition, prototype representations occurred in the right ventral inferior frontal gyrus (IFG; Table 2). Again, there was no evidence for prototype representations in the anterior hippocampus (largest effect at *x* = −18, *y* = −18, z = −22; *Z* = −0.22, *p*_unc_ = .558, *p*_FWEROI_ n.s.) or the vmPFC (largest effect at *x* = −8, *y* = 56, *z* = 8; *Z* = 2.00, *p*_unc_ = 0.023, *p*_FWEROI_ n.s.).

**Table 2:** Localisation of multivariate prototype and exemplar neural representations.

		Peak voxels and local maxima					Cluster extent
		MNI coordinates					
		x	y	z	Z	*p*_FWE_	*k*_FWE_
Prototype model							
Lateral superior OG, IPS (Rostrodorsal IPL, caudal SPL)	R	28	−62	40	5.14	0.001	778
Lateral superior OG, IPS (Rostrodorsal, caudal and rostroventral IPL, caudal SPL)	L	−36	−70	40	4.81	0.006	492
		−26	−60	38	4.38	0.032	
		−30	−76	46	4.33	0.040	
Lateroventral FG, ventrolateral ITG	R	46	−52	−8	4.52	0.019	43
IPS (lateral SPL)	R	28	−42	40	4.42	0.027	52
Ventral IFG	R	51	9	18	4.34	0.039	13
IPS (rostroventral IPL)	R	52	−60	38	4.33	0.039	25
Exemplar model							
Dorsal and caudal cingulate gyrus, paracentral lobule	L/R	2	−27	44	5.07	0.002	783
		−8	−24	51	5.04	0.002	
IPS (rostroventral IPL), postcentral gyrus, superior temporal gyrus	L	−51	−22	16	4.63	0.011	150
Dorsolateral putamen, dorsal insular gyrus	L	−36	−3	3	4.5	0.018	92
Postcentral gyrus, precentral gyrus	R	32	−26	56	4.44	0.023	106
Dorsolateral putamen, dorsal insular gyrus	R	33	4	3	4.4	0.028	52
Postcentral gyrus	L	−38	−27	44	4.36	0.033	45
Anterior hippocampus	R	36	−10	−20	4.34	0.035	11

*OG* occipital gyrus, *IPS* intraparietal sulcus, *IPL* inferior parietal lobule, *SPL* superior parietal lobule, *FG* fusiform gyrus, *ITG* inferior temporal gyrus, *IFG* inferior frontal gyrus.

Peak voxels and local maxima for fits of the prototype and exemplars models surviving family-wise correction for multiple comparisons across the entire scan volume (*p*FWE <0.05, *k* ≥10).

Multivariate exemplar representations were present in parietal, (medio-)temporal and frontal regions (see Fig. 3b and Table 2), including the left IPS, the right anterior hippocampus and the vmPFC (*x* = 10, *y* = 38, *z* = −30; *Z* = 4.21, *p*_FWEROI_ = 0.028). Temporal clusters covered the superior temporal gyrus (Table 2), the rostral ITG (*x* = −38, *y* = 0, *z* = −40; *Z* = 4.28, *p*_FWEROI_ = 0.028) and the ventral ITG (*x* = 45, *y* = −12, *z* = −30; *Z* = 4.13, *p*_FWEROI_ = 0.038). In addition, exemplar representations were present in the bilateral putamen and the insular gyrus (see Fig. 3b and Table 2).

Bayesian analyses were used to quantify the probability by which multivariate effect sizes (ES_RSA_) in bilateral anatomical masks deviate from zero (Fig. 3c, Supplementary Table 2 and Supplementary Note 2). Confirming results from our frequentist analyses, Bayesian analyses provided evidence for multivariate prototype or exemplar representations in different regions. Specifically, Bayesian analyses revealed (1) moderate evidence for prototype representations in the lateral SPL and the inferior frontal sulcus of the IFG and very strong evidence in the rostrodorsal IPL with at least very strong for the absence of exemplar representations in these regions; (2) strong evidence for exemplar representations in two ITG regions, with moderate to strong evidence against prototype representations in the ventral ITG but inconclusive results regarding the lateral ITG; (3) moderate evidence for exemplar representations in the anterior hippocampus and very strong evidence in posterior hippocampus, with moderate evidence for the non-presence of prototype representations in hippocampal; (4) extreme evidence for the absence of prototype representations and moderate evidence for the absence of exemplar representations in the vmPFC. The difference between the latter result and frequentist analyses is likely related to the small extent and pronounced lateralisation of exemplar representations in this region.

Fig. 3d depicts violin plots of individual ES_RSA_ from ROIs with at least moderate evidence for prototype or exemplar representations or being of particular interest.

### Co-existence of multivariate prototype and exemplar representations within individuals

Region-of-interest RSA analyses on the individual level were employed to investigate the potential co-occurrence of prototype and exemplar representations within individuals and their relation to behavioural strategy preferences.

**Fig. 4:**
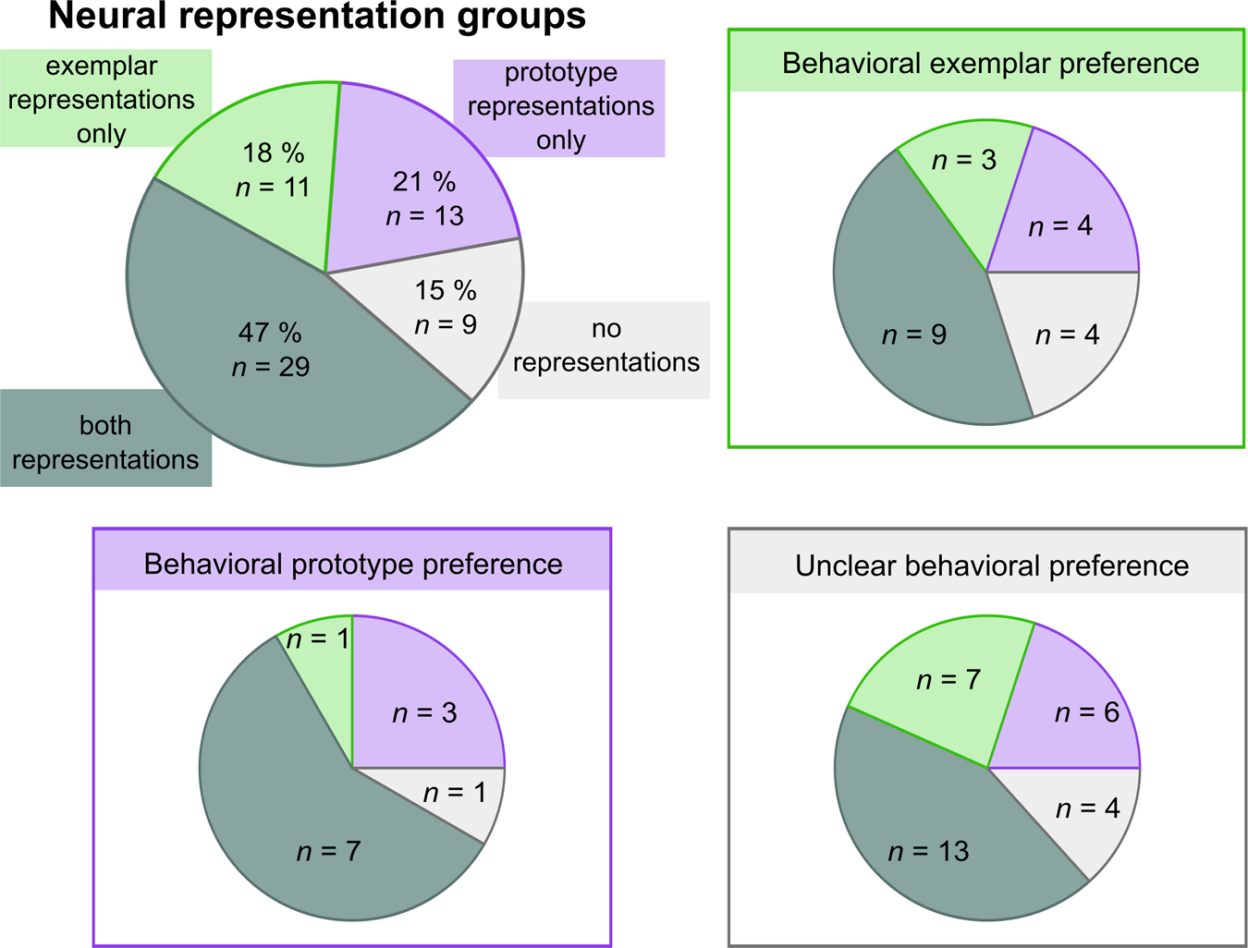
Neural representation groups organised by behavioural preference groups (*n* = 62 individuals) based on ΔDIC ≥5. The pie charts depict the proportion of individuals showing only exemplar representations in green, only prototype representations in purple, the presence of both representations in dark turquoise, and the presence of neither representation type in grey. Supplementary Fig. 1 additionally depicts the subgrouping of neural representation groups when no threshold for the ΔDIC is applied.

In line with previous reports of co-existent prototype and exemplar representations on the group level, current RSA analyses on the individual level indicated the co-occurrence of prototype and exemplar representations in 29 out of 62 individuals (i.e., *ρ* ≥ 0.1 and *p*_unc_ < 0.05 for at least one ROI), 13 individuals showed only prototype and 11 individuals only exemplar representations. A total of nine individuals did not show any representation exceeding the predefined threshold. Figure 4a illustrates that the relationship between neuronal representation groups (i.e., only prototype, only exemplar, both or no representations) and behavioural preference is inconclusive (see also Supplementary Fig. 1).

Importantly, the co-occurrence of the two representation types was not caused by higher correlations between prototype and exemplar model RDM’s in individuals showing evidence for co-existent representations (*M*_*ρ*_ = 0.178; SD_*ρ*_ = 0.051) compared to the 23 individuals who show only evidence for one particular model (*M*_*ρ*_ = 0.176; SD_*ρ*_ = 0.032; *t*(47.75) = 0.22, *p* = 0.825). In summary, current results provide evidence that the two representation types can co-exist within individuals.

## Discussion

We show that prototype representations in a single-category categorisation task with abstract visual patterns are primarily present in visual and parietal areas, with Bayesian analyses yielding a high probability for the non-existence of prototype representations in the hippocampus and the vmPFC. In line with the assumption that different cognitive mechanisms underlie prototype- and exemplar-based categorisation, prototype and exemplar representations were localised in different regions. Moreover, brain activity correlated exclusively negatively with model-based similarity to the prototype, whereas correlations with similarity to exemplars were both positively and negatively signed. Multivariate analyses additionally revealed exemplar representations in memory-related regions like the hippocampus and the inferior temporal gyrus. Furthermore, we report evidence for the co-existence of prototype and exemplar representations on the individual level, indicating that individuals can from prototype and exemplar representations simultaneously.

We used a twofold approach to localise exemplar- and prototype-dependent representations by (1) correlating perceptual similarity to exemplars and the prototype with univariate fMRI activity and (2) comparing multivariate pattern similarity between items based on their perceptual similarity and dissimilarity to exemplars and prototype. The univariate parametric contrast revealed regions in which overall activity to a stimulus scaled positively or negatively with its model-based similarity to the prototype or exemplars, respectively^27^. In addition, the RSA searchlight enabled the localisation of brain regions in which activation and deactivation across voxels contribute simultaneously to the similarity of neural activity patterns between any two items depending on the degree to which model-based similarity to the prototype or exemplars of the two stimuli resemble each other.

Insight into potential processes underlying the prototype and exemplar strategies is given by the inspection of the sign of the correlation between perceptual similarity and univariate fMRI activity. Only perceptual similarity to exemplars but not to the prototype correlated positively with univariate fMRI activity, suggesting qualitatively different mechanisms mediating the two strategies. While both strategies are based on the comparison of an item to a stored representation, exemplar-based categorisation requires an additional active, serial retrieval of single exemplars from memory^4^. This is also supported by the positive correlation with perceptual similarity to exemplars in posterior occipital areas, known to mediate basic visual recognition memory^28^ as well as in the fusiform gyrus, mediating object recognition^29,30^ and the retrieval of visual features^31^. Negative correlations with perceptual similarity, i.e., perceptual dissimilarity (Fig. 2a), occurred for both prototype and exemplar models, albeit in different regions. This negative correlation is in line with expectation suppression^32,33^ and predictive coding theories^34^, assuming that the brain computes the deviation between sensory input and prior representations. Accordingly, activity in visual areas might scale with the deviation from exemplars and the prototype reflecting fine-grained information^35–39^. To summarise, univariate analyses indicate that while the comparison of an item to the prototype mostly reflects its deviation from a holistic visual representation, the comparison to exemplars is mediated by both the processing of perceptual deviation and memory-related processes in which exemplars are actively retrieved and matched to the input.

RSA revealed additional prototype and exemplar representations in inferior parietal and frontal regions, consistent with previous work showing that multivariate voxel patterns in these regions reflect behaviourally relevant category distinctions^27,40^ and information pattern reinstatement during categorisation^41^. Multivariate prototype representations were additionally present in superior parietal regions, which are (1) involved in perceptual evidence accumulation^42,43^ and (2) areas of convergence of multiple perceptual processing streams, enabling the progressive abstraction of conceptual knowledge from perceptual experience^44^. Activity in parietal and prefrontal areas has also been related to difficulty, uncertainty, and related variables (e.g., response conflict resolution or decision-bound implementation)^45,46^. Hence it would be interesting to investigate to what degree parietal and frontal category representations also reflect the recruitment of executive functions inherent in categorisation processes.

The current Bayesian analyses provided moderate to strong evidence that prototype representations were not present in hippocampal areas in this perceptual single-category categorisation task, for which previous studies found relatively preserved performance in patients with memory deficits and hippocampal damage as well as a predominant engagement of the perceptual learning system in healthy individuals^19–22^. The apparent contradiction to reports of prototype representations in the hippocampus of healthy individuals during two-category tasks with stimuli with clearly identifiable binary features could hence be reconciled by hippocampal prototype representations depending on task and stimulus characteristics. The idea of a task- and stimulus-specific hippocampal involvement in prototype representations also fits well to research focusing on categorisation in general: where hippocampal contributions to categorisation have been shown to depend on whether one or two categories are present^14,15^, incidental versus intentional learning modes^47,48^, category structures^49^, as well as learning stage^50^. In addition, a stronger hippocampal involvement in the categorisation of stimuli with clearly identifiable dimensions compared to stimuli like dot patterns is consistent with the central role of the hippocampus in relational binding^11,51^. Although neural prototype representations for A/non-A tasks with dot-pattern stimuli have not yet been addressed directly, more direct evidence for the task- and stimulus-dependency of hippocampal contributions to prototype-based categorisation comes from studies in memory-impaired individuals who showed reduced performance in a two-category but not single-category task^22^ and for the categorisation of scenes but not faces^19^. Since these patients could not rely on the memory-intense exemplar strategy^52,53^, they likely preferred the ‘memory-friendly’ prototype strategy. As prototype representations in tasks with stimuli with clearly identifiable dimensions from two categories depend on hippocampal areas^6,7^, patients were less impaired in single-category tasks in which stimuli can be processed in a holistic manner, such as faces or abstract patterns.

We found multivariate exemplar representations in areas mediating episodic memory, mental search, and/or mental replay, such as the vmPFC, the putamen, the cingulate gyrus, the insula, and most notably, the anterior and posterior hippocampus^54–58^. Hippocampal exemplar representations support the view that the exemplar strategy involves the recall of single stimulus instances^59,60^. Previous work suggests that the anterior hippocampus is more involved in associative processes, whereas the posterior hippocampus mainly mediates distinct representations supporting stimulus discrimination^11^. We found both pronounced posterior hippocampal exemplar representations in line with previous literature using simple exemplar models^7^ and anterior hippocampal exemplar representations, that were so far only demonstrated in previous studies using more sophisticated models^55,61^ like SUSTAIN that build adaptive clusters varying in their degree of abstraction from exemplar-like to prototype-like representations. Therefore, the detection of exemplar representations in the anterior hippocampus might require relatively large sample sizes as in our study or more sophisticated modelling approaches as in previous studies.

Inspection of multivariate neural representations within individuals supports the view that prototype and exemplar representations can be stored simultaneously (Fig. 4). A similar phenomenon has been described for recognition memory, where the same events and objects were represented simultaneously at different resolutions^62,63^, and for decision making, where value estimates were simultaneously represented based on recent but also long-term experience^64^. The formation of both representation types, i.e., prototype as well as exemplars, might preserve high flexibility in behavioural strategies so that categorisation could be based either on prototype or exemplar representations, depending on the current stimulus properties and/or resources. Here, we used an exploratory descriptive approach for describing prototype and exemplar representations on the individual level so that the exact proportion of individuals using both representations warrants support from future research.

The current article focused on the localisation and co-existence of prototype and exemplar representations in the brain. Connecting to previous research using a similar approach^6–8^, we used simple prototype and exemplar models instead of more complex models such as mixture models or SUSTAIN. In addition, reflecting that comparing a new item to a relatively large set of individual exemplars involves more complex cognitive processes than the comparison to a single prototype, similarity estimates produced by the exemplar model are naturally more complex than those from the prototype model, which in turn poses the risk that smaller exemplar-dependent effects could have been overlooked. Nevertheless, our approach confirmed exemplar representations in previously described regions. Particularly the localisation of (more complex) exemplar representations in the hippocampus reinforces our findings of non-present hippocampal prototype representation in the current task. Relatedly, it should be noted that prototype and exemplar models can be differentially flexible to fit the data^65^, however in the present study, both strategies were identified. Moreover, although the non-presence of hippocampal prototype representations in the current results could provide a good explanation for relatively preserved categorisation performance in single-category tasks with abstract visual patterns in memory-impaired individuals, they do not rule out general hippocampal contributions to prototype-based perceptual categorisation. Furthermore, it would be interesting to test whether the similarity structure of prototype and exemplar representations identified in our data corresponds to the perceived similarity of our stimulus set, as suggested by previously reported high correspondence between objective and subjective similarity measures^13,66^. Finally, although significant learning effects during training speak against a strong contribution to this issue in our task, it is worth noting that A/notA tasks pose the risk of reaching above-average categorisation without training^67^.

In summary, our approach to measuring univariate and multivariate exemplar and prototype representations demonstrates that prototype representations in a single-category task with abstract visual patterns were predominantly mediated by visual and parietal but not hippocampal areas. This finding could account for apparent contradictions between hippocampal prototype representations in two-category tasks and relatively preserved prototype-based categorisation in individuals with hippocampal lesions in single-category tasks. In particular, we provide evidence that prototype representations in single-category perceptual categorisation with abstract visual stimuli are mainly formed based on category-relevant deviations from the prototype in visual areas like the fusiform gyrus. Exemplars seem to be represented as fine-grained memory representations in visual areas, with memory-based evidence for category membership being further processed in memory-related areas like the hippocampus. Moreover, our results indicate that individuals can form both representation types simultaneously.

## Methods

### Participants

A total of *N* = 72 volunteers took part in the study. Volunteers were recruited via advertisement on a job board of the University of Hamburg. Volunteers were eligible to participate when they were between 18 and 35 years old, fluent in the German language, had no history of mental or neurological disease, drug or alcohol abuse and did not have any contraindications to MRI measurement (e.g. metal implants, pregnancy). Moreover, all volunteers were required to have normal or, through contact lenses, corrected-to-normal vision. All gave signed informed consent according to the Declaration of Helsinki. Ethics approval was obtained from the Ethics Committee of the Hamburg Medical Association (PV5874).

Two volunteers felt uncomfortable in the scanner, one measurement was interrupted by technical problems and one volunteer was measured with wrong sequence settings. Datasets from these volunteers were excluded from all analyses. In addition, volunteers were excluded who both performed below 75% during the last training block of the category learning task and did not reach an average performance of 75% during the test phase (*N* = 5). The data from an additional volunteer were excluded because the fit of the guessing model did not show a meaningful difference between the fits of the prototype and the exemplars models (see Methods Section ‘Computational modelling’).

In total, *N* = 62 volunteers were included in further analyses. Included volunteers were between 19 and 34 years old [*M* = 25.6, SD = 3.7 years]. *N* = 30 of the volunteers were female and *n* = 8 were left-handed. Volunteers had at least 12 years of education, with the majority being currently enroled as university students (*n* = 38).

### Stimuli

Dot patterns were created using a well-established procedure originally published by Posner and colleagues^13^. The prototypical stimulus of category A was created by placing nine dots at random positions within the central 30 × 30 area of a 50 × 50 grid. Category members were created by moving each dot of the prototypical stimulus probabilistically following a procedure published by Posner and colleagues (1976), in which varying levels of distortions from the prototype are implemented by the application of different probability sets for dot movement. These probability sets determine whether a dot will either keep its position or the distance by which it will be moved within a 20 × 20 around its original position. For the training set, four stimuli with distortion levels of 5.0, 6.0 and 7.0 bit/dot were randomly chosen as exemplars, summing up to 12 idiosyncratic category members. Non-member training stimuli were 12 randomly generated dot patterns with dot positions located within the central 30 × 30 area of a 50 × 50 grid, each pattern was distorted by a level of 7.0 bit/dot to prevent non-members to be easily identified by a more central positioning of the dots compared to the distorted category members. As in the classical paradigm, the prototype was not part of the training set. The transfer set consisted of the prototype, the 24 stimuli from the training set, 8 novel distorted versions of the prototype (distortion levels of 4.0, 5.0, 6.0 and 7.0 bit/dot) and eight novel distorted versions of random patterns (distortion level of 7.0 bit/dot). Following a common practice^4,45,66,68^, dots were connected to polygons to make the stimuli visually more appealing and facilitate holistic processing.

### Category learning task

Participants were informed that they would learn via trial-and-error whether abstract patterns belong to a certain category (‘A’) or not. They were explicitly instructed to focus on the overall appearance of each stimulus and the similarity among category members. They were informed that no single feature was informative of category membership and that there was no hidden verbalisable rule. Before the acquisition of the actual category structure, two familiarisation blocks consisting of 24 trials with a different stimulus set of dot-pattern stimuli ensured that volunteers understood the task.

Participants performed the acquisition and the transfer phases of the categorisation task in the MRI scanner. Each training block consisted of all 24 stimuli from the training set (12 ‘A’ & 12 ‘non-A’ stimuli), presented individually in pseudo-randomised order at a visual angle of 4.22 degrees. Volunteers had to indicate via button press within a time window of 2.5 s whether they believed a pattern belongs to category A or not. The position of ‘yes’ and ‘no’ responses on the screen changed pseudo-randomly between left and right to prevent stimulus-response mapping. Visual feedback was presented after each trial indicating whether the volunteers’ response was ‘correct’, ‘incorrect’, or ‘too late’. Total accuracy per block was shown after each training block. All participants performed at least eight training blocks. Training continued until completing four blocks with an accuracy of 75% or after 14 training blocks.

During the acquisition phase, each fMRI run comprised three training blocks, giving individuals the opportunity to take a short break within the scanner after every third block. To minimise fatigue, all participants left the scanner after the acquisition phase (~23 to 40 min) for a period of 5 to 10 min. During the transfer phase, each block consisted of 24 training stimuli plus 17 novel stimuli (the prototype, eight distorted ‘A’ members and eight ‘non-A’ stimuli). Stimuli were presented in pseudo-randomised order without feedback. To optimise fMRI acquisition for multivariate fMRI analyses, individual fMRI runs were used for the 8 transfer blocks (each ~4.8 min). See Fig. 1b for details on trial timing.

Because the research questions of the current articles focus on stable prototypes and exemplar representations, only data from the transfer phase were included in further analyses.

### Computational modelling

Classical A/non-A prototype and exemplar models^60,69,70^ were applied to endorsement rates of the transfer phase to study prototype and exemplar representations in the brain (Fig. 1b, c). Both models define perceptual similarity (*s*) as an exponential decay function with a sensitivity parameter (*c*), quantifying the steepness of how perceptual similarity decays with distance (*d*). The prototype model postulates that the similarity of item *i* to category A is represented by its similarity to the prototype *P* (i.e. *s*_iA_ = ${s}_{{{iP}}_{A}}$). The exemplar model postulates that the similarity of item *i* to category A is represented by the summed similarity to training exemplars *j* ($i.e.,{{s}}_{{iA}}=\mathop{\sum}\limits_{j\in A}{{s}}_{{ij}}$).

1 $${{{{{\rm{similarity}}}}}}\; {{{{{\rm{of}}}}}}\; {{{{{\rm{the}}}}}}\; {{i}}{\mbox{-}}{{{{{\rm{th}}}}}}\; {{{{{\rm{item}}}}}}\; {{{{{\rm{to}}}}}}\; {{{{{\rm{prototype}}}}}}:{s}_{{iA}}={e}^{-c{d}_{{iP}}}$$

2 $${{{{{\rm{similarity}}}}}}\; {{{{{\rm{of}}}}}}\; {{{{{\rm{the}}}}}}\; {{i}}{\mbox{-}}{{{{{\rm{th}}}}}}\; {{{{{\rm{item}}}}}}\; {{{{{\rm{to}}}}}}\; {{{{{\rm{exemplars}}}}}}\; {{{{{\rm{j}}}}}}:{s}_{{iA}}=\mathop{\sum}\limits_{j\in A}{e}^{-c{d}_{{ij}}}$$

While physical similarity is based on Euclidian distances (*d*) between stimuli, perceptual similarity (*s*) is derived using an exponential function in which physical distances are scaled by an individual sensitivity parameter (*c*) taken to the power of the Euler’s number (1/2). Accordingly, physical distances farther away from the prototype or exemplars are down-weighted, with the amount of down-weighting being determined by individual sensitivity. The resulting estimates of perceptual similarity have been shown to closely match similarity ratings in previous studies^13,66^.

The endorsement probabilities (*r*) are based on the similarity to category A (*s*_A_) plus a criterion parameter (*k*).

3 $${{{{{\rm{endorsement}}}}}}\; {{{{{\rm{probability}}}}}}\; {{{{{\rm{of}}}}}}\; {{{{{\rm{the}}}}}}\; {{i}}{\mbox{-}}{{{{{\rm{th}}}}}}\; {{{{{\rm{item}}}}}}:{r}_{i}=\frac{{{s}}_{{iA}}}{{{s}}_{{iA}}+\,k}$$

Fitting of the prototype and exemplar models was performed using the package ‘R2jags’^71^ under R version 4.0.3^72^ which implements Bayesian analysis in JAGS^73^. To identify volunteers whose performances could be better explained by guessing than by a prototype or exemplar strategy^74^, a guessing model was estimated assuming endorsement probability to be at chance level (i.e. *r* = 0.5). All three models were fit to endorsement rates of the transfer phase individually for each volunteer. Uniform priors were used for sensitivity [*c* ~ uniform(0,5)] and criterion parameters [*k* ~ uniform(0,1)] of the prototype and exemplar models, representing beliefs before fitting the models to the data that c varies between 0 and 5 and k between 0 and 1 with homogenous probabilities. Markov Chain Monte Carlo (MCMC) algorithms provided by the JAGS software were used for parameter estimation. Starting values for parameter estimation were created by first fitting the two models without initial value definition (4000 repetitions, burn-in period of 1000 repetitions, four chains) and then using the 2.5, 25, 50, 75 and 97.5% quantiles of the posterior distributions (i.e. the updated beliefs after model fitting to the data) as initial values for the next modelling step. The final model estimation was performed using 100,000 repetitions, a burn-in period of 25,000 repetitions and four chains. To reduce autocorrelations, a thinning factor of 5 was used so that only every fifth sample from the posterior distribution was kept. Chain convergence diagnosis, i.e., testing whether the simulated draws reached its stationary state, was performed by visual inspection and by calculating the potential scale reduction factor (Rhat)^75^. The JAGS software provides the Deviance information criterion (DIC)^26^ as a default measure for Bayesian model fit, a hierarchical modelling generalisation of the Akaike Information Criterion (AIC). Following the previous literature^76–78^, a ΔDIC ≥5 was interpreted as a threshold for a meaningful difference. Posterior means of posterior distributions for the two free parameters were used for subsequent analyses, including the generation of trial-wise similarity vectors for each individual volunteer [formula (1) and (2)].

Only similarity estimates of category members were used as parametric modulators in univariate fMRI analyses and for the generation of model RDMs for multivariate fMRI analyses for two reasons. Firstly, the fact that category members have a higher average similarity to the prototype/exemplars than non-member stimuli would introduce the main effect of category membership to the similarity vectors. Secondly, prototype and exemplar similarity vectors of non-members were highly correlated (*M*_*r*_ = 0.991, SD_r_ < 0.01) and are therefore not informative to distinguish prototype and exemplar representations. This was not the case for similarity vectors of category members (*M*_*r*_ = −0.249, SD_r_ = 0.08).

### Regions of interest (ROI)

Region-of-interest definition was based on the three existing model-based fMRI studies on prototype and exemplar representations using complex categorisation tasks^7,8,11^ as well as on a study using a basic perceptual categorisation task which did not directly target prototype/exemplar representations^45^. We selected the following regions of interest: (1) prefrontal regions^7,8,11^, specifically the IFG and the vmPFC; (2) parietal regions^8,11,45^, such as the IPS [in this atlas labelled as rostral, caudal and lateral SPL and rostrodorsal inferior parietal cortex (IPL)] and the precuneus; (3) occipital areas^8^ and occipito-temporal regions^45^, specifically, the ITG and the fusiform gyrus; and (4) the hippocampus^6,7^.

All masks were created by using the BRAINNETOME atlas (BNA)^79^. Additionally, a mask for the vmPFC was taken from Mackey and Petrides^80^. Since the BNA provides a different division of the hippocampus than used by Bowman and colleagues^6^, hippocampal masks extracted from the atlas were first collapsed and then divided at the middle slice into an anterior and a posterior division. In total, six IFG masks, seven ITG masks, three fusiform gyrus masks, nine parietal regions masks, 11 occipital regions masks, two hippocampus and one vmPFC mask covering both hemispheres (see Supplementary Table 3 for BNA IDs) were selected. For family-wise error correction of uni- and multivariate analyses on the group level, the 39 masks were combined into a single ROI mask.

### Image acquisition and preprocessing

Event-related functional MRI was performed on a 3 Tesla scanner (Siemens PRISMA) with a multiband gradient echo-planar imaging T2*-weighted sequence in 54 contiguous axial slices (2-mm thickness, TR 1.636 s, TE 29 ms, flip angle 70°, a field of view 224 × 224, multiband factor 2). For spatial normalisation, a high-resolution T1-weighted structural MR image was acquired by using a 3D-MPRAGE sequence (TR 2.3 s, TE 2.89 ms, flip angle 9°, 1 mm slices, a field of view 256 × 192; 240 slices).

Neuroimaging data were preprocessed and analysed using Statistical Parametric Mapping (SPM12; Wellcome Department of Imaging Neuroscience, London, UK) run in Matlab R2020b. To prevent biases due to spin saturation, the first five functional images were discarded. To correct for susceptibility-by-movement artefacts, all functional images were realigned and unwarped (as implemented in SPM12). Individual structural T1 images were coregistered to functional images, segmented into grey and white matter and submitted to the ‘diffeomorphic anatomic registration through an exponentiated lie algebra algorithm’ (DARTEL) toolbox to create structural templates, individual flow fields as well as subject-specific grey matter, white matter and cerebrospinal fluid (CBF) masks. DARTEL flow fields were used for normalising structural and functional images to MNI space.

We conducted several first-level models (for different versions, see next sections) for which functional images from the transfer phase were submitted to general linear models (GLMs) with a 128 s high pass filter. We applied SPM’s alternative pre-whitening method to account for autocorrelation, FAST, which has been suggested to perform better than SPM’s default^81^.

### Univariate fMRI analyses

For univariate group-level analyses, normalised functional images were smoothed with a full-width half maximum (FWHM) Gaussian kernel of 8 mm in all spatial directions. The kernel size of 8 mm was chosen based on our previous work targeting cortical and hippocampal regions^82,83^, the observation that larger smoothing kernels can increase sensitivity to hippocampal activity^84^ and general recommendations^85^. To make sure that we did not overlook effects in small hippocampal regions due to an 8 mm smoothing kernel, we repeated the univariate analyses on functional images smoothed with 4 mm (see Supplementary Note 2). White matter and CBF masks were used to extract time series representing noise unrelated to the experimental paradigm. Principal components explaining at least 1% of variance were used as nuisance regressors in all first-level models.

Two GLMs were estimated for each subject to localise univariate prototype and exemplar representations. For each GLM, the eight runs from the transfer phase were concatenated and a session constant was added. GLMs contained an onset regressor for correctly categorised category members modulated by trial-wise prototype or exemplar similarity estimates in two separate GLMs, respectively. To explain the additional variance, onsets for correctly classified non-members, onsets for incorrectly categorised patterns (members and non-members), response onsets modulated by reaction times, onsets of fixation crosses, as well as nuisance regressors were added as regressors of no interest.

Univariate prototype and exemplar representations for each individual were measured by contrasting BOLD effects associated with the parametric modulator containing prototype or exemplar similarity estimates for correctly classified category members against the implicit baseline. The resulting contrast images were submitted to one-sample *t*-tests. Results were considered significant at *p* < 0.05, family-wise error corrected for multiple comparisons on the entire scan volume (*p*_FWE_) and within the regions of interest mask (*p*_FWEROI_), and an extent threshold of ten contiguous voxels.

In addition to assessing localisations of prototype and exemplar representations using classical peak-based frequentist approaches, we applied a Bayesian analysis approach. This approach provides an opportunity to quantify the probability of the (non-)existence of prototype and exemplar representations in specific brain areas on the group level and thereby also connects our findings to previous research focusing on ROI-based group analyses^6^. For this purpose, Bayesian analyses were conducted using the brm() function from the R-package ‘brms’. To calculate ROI-specific effect sizes (ES_univ_)^6^ for each individual, contrast estimates from each of the 39 bilateral masks associated with univariate prototype and exemplar representations were averaged and then divided by the standard deviation. Next, individual ROI-specific ES_univ_ were submitted separately to simple intercept-only models (i.e., ES_univ_ ~0 + intercept) as well as models assuming an intercept of zero (i.e., ES_univ_ ~0). Model estimation was performed using a uniform prior distribution [ES_univ_ ~uniform(−5,5)], 40,000 repetitions, a burn-in period of 2500 repetitions and four chains. Following the interpretation of evidential strength of the standards proposed by Jeffreys^86^ and adapted by Lee and Wagenmakers^87^ (*p*. 105), a Bayes factor (BF) >3 was interpreted as evidence that ES_univ_ deviates from 0 (H_1_) and a BF <0.333 as evidence that ES_univ_ was sampled from a null distribution (H_0_).

### Multivariate fMRI representational similarity analyses (RSA): first-level models and generation of representational dissimilarity matrices (RDM)

We performed two RSA analyses. (1) To identify multivariate prototype and exemplar representations on the group level in a whole-brain searchlight analysis, realigned native-space functional images from the transfer phase were submitted to subject-specific GLMs. (2) To identify multivariate prototype and exemplar representations in individual ROIs (located in MNI space) within single individuals and relate them to behavioural model fits, normalised functional images from the transfer phase were submitted to subject-specific GLMs.

For our design with jittered ITI and randomised order of conditions within and across runs, the best modelling approach is to model the data with a regressor for all of the trials within the condition of interest^88^. This approach is optimal for obtaining stable estimates of activity for each condition because the model is fit for multiple observations. Hence, individual GLMs contained separate regressors for all 41 stimuli, each holding eight onsets reflecting stimulus repetitions. Similar to the first-level models for univariate analyses, regressors containing response onsets, modulated by reaction times, and onsets of fixation crosses, as well as nuisance regressors, were added as regressors of no interest.

We used the resulting single stimulus *t*-maps of category members (i.e., 21 *t*-maps) for RSA using the RSA-toolbox^89^. *T*-maps were chosen because they consist of effect sizes weighted by their error variance, which reduces the influence of large but variable response estimates for multivariate analyses^90^. In general, RSA involves testing whether the observed similarity of brain responses (a neural RDM) corresponds to a hypothetical pattern of similarity (model RDM).

To generate the model RDMs, trial-wise estimates of similarity to the prototype and exemplars of category members were first rescaled. Each cell of the model RDMs, representing one stimulus pairing, was then filled with the absolute difference in model-based similarity estimates for each stimulus pair (Fig. 3a). In contrast to the above-mentioned parametric modulators directly testing whether BOLD effects correlate with model-based perceptual similarity or dissimilarity to the prototype/exemplars, the multivariate RDMs measure how closely stimuli resemble each other regarding their model-based similarity to the prototype or exemplars. In the univariate approach, stimuli with low similarity to the prototype have a smaller value for the parametric modulator than stimuli with high similarity to the prototype. In contrast, in our RSA approach, a stimulus has a small RDM-value when being paired with a stimulus that is similar in similarity to the prototype (i.e. when the paired stimuli have both low or both high similarity to the prototype, respectively) and a large RDM-value when being paired with a stimulus that is dissimilar in its similarity to the prototype (i.e. when comparing two stimuli with low and high similarity to the prototype). The difference between the current univariate and RSA approach is that the univariate approach identifies regions in which evoked activity scales with an item’s (dis-)similarity to the prototype or exemplars, whereas the RSA approach identifies regions in which voxel pattern similarity between two items correlates with their resemblance in (dis-)similarity to the prototype or exemplars. Illustrating the difference between the current RSA and other RSA approaches, highly distorted stimuli can look very different but still resemble each other closely in their (dis-)similarity to the prototype or exemplars. Intra-individual Spearman rank correlation coefficients for the relationship between prototype and exemplar RDMs were on average *M*_*ρ*_ = 0.18 (SD_*ρ*_ = 0.041).

We measured neural multivoxel RDMs by computing the dissimilarity (1–Pearson correlation across voxels) of *t*-maps for each specific category member.

### Searchlight RSA (group level)

To assess neural prototype and exemplar representations on the group level across the whole brain using a searchlight approach, the 21 single stimulus *t***-**maps in native space were submitted to RSA searchlight separately for each volunteer. For each searchlight (eight voxels radius), neural RDMs were generated by extracting stimulus-specific *t*-values from *t*-maps masked by individual grey matter masks (thresholded at an intensity value of 0.2). For each subject, neural and model RDMs were compared using Spearman’s rank correlation coefficient. The resulting *ρ*-maps were Fisher *Z*-transformed to conform to Gaussian assumptions, smoothed with an FWHM Gaussian kernel of 8 mm in all spatial directions, normalised using individual flow fields from the DARTEL toolbox, and masked by a normalised, average cerebrum grey matter mask. Again, we repeated the analysis with 4 mm smoothing to make sure we do not overlook effects due to an 8 mm smoothing kernel. Preprocessed *ρ*-maps were submitted to one-sample *t*-tests. We tested for positive correlations between the neural and model RDMs to identify regions in which multivariate pattern activity reflects prototype or exemplar-dependent representations so that activity patterns evoked by two stimuli with similar distances to the prototype (or exemplars) should be more similar than activity patterns evoked by two stimuli with a dissimilar distance to the prototype (or exemplars). Results were considered significant at *p* < 0.05, family-wise error corrected for multiple comparisons on the entire scan volume (*p*_FWE_) and within the regions of interest mask (*p*_FWEROI_), and an extent threshold of 10 contiguous voxels.

As for contrast estimates from univariate analyses, Bayesian analyses were conducted on ROI-specific averages of correlation coefficients from normalised *ρ*-maps to quantify the probability that anatomical brain regions carry prototype and/or exemplar representations on the group level. Model estimation on ES_RSA_ was performed using a uniform prior distribution ranging between −1 and 1 [ES_RSA_ ~ uniform(−1,1)].

### ROI RSA (individual level)

To assess the potential co-occurrence of neural prototype and exemplar representations within individuals and study how they are linked to behavioural preferences, the 21 single stimulus *t***-**statistic maps in standard space were submitted to RSA separately for each volunteer within the above-mentioned predefined ROI^89^. For each ROI, neural and model RDMs were compared using Spearman’s rank correlation coefficient. Given the large number of ROIs, uncorrected statistical comparisons would yield a high chance of false positives, while a Bonferroni correction for multiple comparisons is likely associated with a decreased sensitivity. Therefore, we relied on the correlation coefficients as effect sizes and uncorrected *p* values of the condition-label randomisation test of RDM relatedness as implemented in the RSA-toolbox^89^, with a threshold of *ρ* ≥ 0.1 (i.e., at least a small effect size^91^, p. 82) and *p*_unc_ < 0.05 being interpreted as evidence for the presence of prototype or exemplar representations. Concerning the relationship between neural and behavioural model fits investigated with Spearman’s rank correlation coefficient analyses, a similar critical threshold of *ρ* ≥ 0.1 and *p*_unc_ < 0.05 was used.

### Analyses of behavioural data

Linear models were estimated using the lm() function to compare accuracy between the three preference groups. Linear mixed models were estimated using the lmer() function of the lme4-package^92^ in combination with the lmerTest-package^93^ to investigate the effects of category membership and training/transfer blocks on accuracy and to test whether model-free distances to the prototype or exemplars predict accuracy. A paired *t*-test was performed using the *t*.test() function to compare the DICs of the two strategies. Results were considered significant at *p* < 0.05.

### Statistics and reproducibility

Please refer to the Methods section for details about the software, analyses and statistics used in the present study. All analyses were based on data from 62 individuals (see Methods Section ‘Participants’).

## Supplementary information

Supplementary Information

## Data Availability

The experimental data that support the findings of this study are available from https://osf.io/xkgqz/?view_only=9b02442f834c44f8b4923996f0e41ff6, including the source data underlying Figs. 2d, 3d placed in a subfolder named ‘Functional imaging analyses’.
