# Supplementary material for: Functional imaging analyses reveal prototype and exemplar representations in a perceptual single-category task
Source: Commun Biol. 2022 Sep 1;5:896. doi: 10.1038/s42003-022-03858-z (PMC9437087; doi:10.1038/s42003-022-03858-z)
Supplement: Supplementary file 1 — Supplementary Information [file 42003_2022_3858_MOESM1_ESM.pdf]

Supplementary Information

Supplementary Note 1: Behavioral performance in the training phase

Individuals performed on average 9.84 training blocks ($SD = 2.33$ blocks). An accuracy level of at least .75 was reached after 5.4 training blocks on average ($SD = 3.5$ blocks). Accuracy significantly increased across training blocks [F(13, 14548.7) = 34.56, p < .001], with no association between learning and category membership [F(13, 14528.2) = .86, p = .601]. In the final training block, individuals had an average accuracy of .88 ($SD = 0.08$). Average response speed was 1349.12 ms ($SD = 405.22$ ms).

Supplementary Note 2: Bayesian analyses of hippocampal prototype representations based on data smoothed with 4 mm

To make sure that the absence of hippocampal prototype representations is not a result of the chosen smoothing kernel, we repeated univariate and multivariate analyses on functional images smoothed with 4 mm and extracted effect sizes (ES) from anterior and posterior hippocampal masks. Consistent with analyses on data smoothed with 8 mm, Bayesian analyses on univariate ES (ES_{univ}) based on data smoothed with 4 mm deliver strong evidence for the absence of prototype representations in the anterior hippocampus (BF = .04) and very strong evidence for the absence of prototype representations in the posterior hippocampus (BF = .01). Similarly, Bayesian analyses on multivariate ES (ES_{RSA}) based on correlation maps smoothed with 4 mm still deliver moderate evidence for the absence of prototype representations for the anterior (BF = .13) and the posterior hippocampus (BF = .17).

Supplementary Table 1: ROI-specific evidence for the presence of univariate prototype and exemplar representations (*n* = 62 individuals)

ROI	prototype model					exemplar model				
	p. prob. for H_1	*BF for H_1*	*ES_{univ} estimate*	*95% CI*		*p. prob. for H_1*	*BF for H_1*	*ES_{univ} estimate*	*95% CI*	
**ROIs with BF > 3 for H_1 for at least one model**										
Caudal LG	.03	.03	.05	-.15	.25	.91	14.97	-.33	-.50	-.15
Lateral OG	.94	22.95	-.38	-.58	-.18	.18	.20	.24	.00	.47
Inferior OG	.85	5.56	-.39	-.62	-.16	.99	81.48	.37	.20	.54
Lateroventral FG	> .99	50753.91	-.43	-.57	-.29	.96	53.80	.32	.17	.47
**Regions of particular interest**										
vmPFC	.02	.02	.02	-.11	.16	.04	.04	.09	-.03	.20
Anterior Hippocampus	.12	.15	-.15	-.31	.00	.05	.05	.09	-.03	.21
Posterior Hippocampus	.02	.02	-.05	-.18	.07	.01	.01	-.02	-.13	.08

Results from Bayesian analyses providing at least moderate evidence for the presence of univariate prototype or exemplar representations (i.e., BF > 3 for H_1) or are with regions of interest (ROIs) of particular interest. BF: Bayes Factor. ES_{univ}: effect size. CI: Credible Interval. LG: Lingual gyrus. OG: Occipital Gyrus. FG: Fusiform gyrus. VmPFC: Ventro-medial prefrontal cortex.

Supplementary Table 2: ROI-specific evidence for the presence of multivariate prototype and exemplar representations (*n* = 62 individuals)

ROI	prototype model					exemplar model				
	p. prob. for H_1	*BF for* H_1	ES_{RSA} *estimate*	*95% CI*		*p. prob. for* H_1	*BF for* H_1	ES_{RSA} *estimate*	*95% CI*	
**ROIs with BF > 3 for** H_1 **for at least one model**										
IF Sulcus	.88	3.70	.03	.01	.04	.01	<.01	.00	-.01	.01
Rostroventral FG	.11	.13	-.01	-.02	.00	.99	16.91	.01	.01	.02
Ventral ITG	.06	.08	-.01	-.02	.00	> .99	20.43	.01	.01	.02
Lateral ITG	.49	.62	-.02	-.02	-.01	.80	4.58	.01	.01	.02
IPS (Rostro-dorsal IPL)	> .99	88.65	.03	.02	.05	.01	.01	.00	-.01	.00
IPS (Lateral SPL)	.69	3.26	.02	.01	.03	.01	.01	.01	.00	.01
**Regions of particular interest**										
vmPFC	.00	< 0.01	.00	-.01	.00	.13	.16	.01	.00	.02
Anterior Hippocampus	.11	.14	-.02	-.03	-.01	.86	4.31	.02	.01	.03
Posterior Hippocampus	.11	.13	-.01	-.02	-.01	> .99	45.06	.02	.01	.02

Results from Bayesian analyses providing at least moderate evidence for the presence of multivariate prototype or exemplar representations (i.e., BF > 3 for H_1) or are with regions of interest (ROIs) of particular interest. BF: Bayes Factor. ES_{RSA}: Multivariate effect size. CI: Credible Interval. IF: Inferior Frontal. FG: Fusiform gyrus. ITG: Inferior Temporal Gyrus. IPS: Intraparietal sulcus. IPL: Inferior parietal lobule. SPL: Superior parietal lobule. VmPFC: Ventro-medial prefrontal cortex.

Supplementary Table 3. ID's of regions of interest extracted from the Brainnetome atlas (BNA)[1]

Regions of interest	BNA IDs L	BNA IDs R	Anatomical description used in the current article
Inferior Frontal Gyrus	29	30	Dorsal Inferior Frontal Gyrus
	31	32	Inferior Frontal Sulcus
	33	34	Caudal Inferior Frontal Gyrus
	35	36	Rostral Inferior Frontal Gyrus
	37	38	Opercular Inferior Frontal Gyrus
	39	40	Ventral Inferior Frontal Gyrus
Superior Parietal Lobe	125	126	Rostral Superior Parietal Lobe
	127	128	Caudal Superior Parietal Lobe
	129	130	Lateral Superior Parietal Lobe
Inferior Parietal Lobe	137	138	Rostrodorsal Inferior Parietal Lobe
	139	140	
Precuneus	147	148	Medial Precuneus
	149	150	
	151	152	Dorsomedial parietooccipital sulcus
	153	154	Precuneus Area 31
Inferior Temporal Gyrus	89	90	Ventral Inferior Temporal Gyrus
	91	92	Lateroventral Inferior Temporal Gyrus
	93	94	Temporal Pole
	95	96	Lateral Inferior Temporal Gyrus
	97	98	Ventrolateral Inferior Temporal Gyrus
	99	100	Caudolateral Inferior Temporal Gyrus
	101	102	Caudoventral Inferior Temporal Gyrus
Hippocampus	215 & 217	216 & 218	Anterior Hippocampus<br>Posterior Hippocampus
Fusiform Gyrus	103	104	Rostroventral Fusiform Gyrus
	105	106	Medioventral Fusiform Gyrus
	107	108	Lateroventral Fusiform Gyrus
Medio-Ventral Occipital Gyrus	189	190	Caudal Lingual Gyrus
	191	192	Cuneus
	193	194	
	195	196	Rostral Lingual Gyrus
	197	198	Ventromedial Parietooccipital Sulcus
Lateral Occipital Gyrus	199	200	Middle Occipital Gyrus
	201	202	Lateral Occipital Gyrus Area V5
	203	204	Posterior Occipital Cortex
	205	206	Inferior Occipital Gyrus
	207	208	Medial Superior Occipital Gyrus
	209	210	Lateral Superior Occipital Gyrus

Supplementary Figure 1: Neural representation groups organized by behavioural preference groups (*n* = 62 individuals) based on the unthresholded ΔDIC

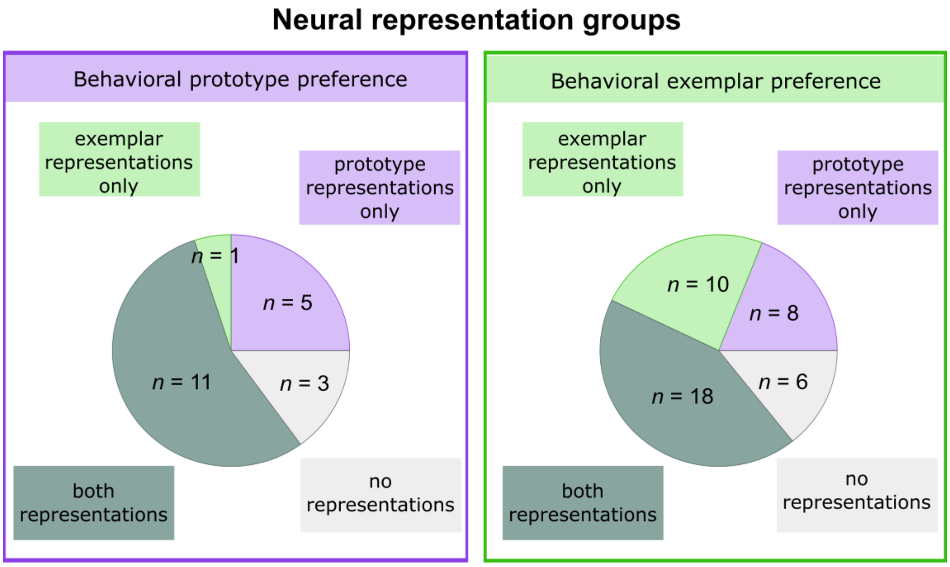

The pie charts depict the proportion of individuals showing only exemplar representations in green, only prototype representations in purple, the presence of both representations in dark turquoise and the presence of neither representation types in grey. Please note that these results should be interpreted with great caution, as differences in DIC's below can be misleading[2].

Supplementary References

1. Fan, L. *et al.* The human brainnetome atlas: a new brain atlas based on connectional architecture. *Cereb. Cortex* **26**, 3508–3526 (2016).
2. Lunn, D., Jackson, C., Best, N., Thomas, A. & Spiegelhalter, D. *The BUGS Book: A Practical Introduction to Bayesian Analysis*. (Chapman and Hall/CRC, 2012).
